# Advances in stromal cell therapy for management of Alzheimer’s disease

**DOI:** 10.3389/fphar.2022.955401

**Published:** 2022-10-04

**Authors:** Rashi Srivastava, Aidong Li, Tirtharaj Datta, Niraj Kumar Jha, Salehikram Talukder, Saurabh Kumar Jha, Zhe-Sheng Chen

**Affiliations:** ^1^ Chemical and Biochemical Engineering, Indian Institute of Technology, Patna, India; ^2^ Department of Rehabilitation, The Second People’s Hospital of Shenzhen, Shenzhen, China; ^3^ Department of Biotechnology, School of Engineering and Technology, Sharda University, Greater Noida, India; ^4^ Institute for Biotechnology, St. John’s University, New York City, NY, United States; ^5^ Department of Biotechnology, School of Applied and Life Sciences, Uttaranchal University, Dehradun, India; ^6^ Department of Biotechnology Engineering and Food Technology, Chandigarh University, Mohali, India; ^7^ Department of Pharmaceutical Sciences, College of Pharmacy and Health Sciences, St. John’s University, New York City, NY, United States

**Keywords:** cellular therapy, mesenchymal stromal cell, Alzheimer’s, clinical trial, management

## Abstract

Deposition of misfolded proteins and synaptic failure affects the brain in Alzheimer’s disease (AD). Its progression results in amnesia and cognitive impairment. Absence of treatment is due to excessive loss of neurons in the patients and the delayed effects of drugs. The enhanced pluripotency, proliferation, differentiation, and recombination characteristics of stromal cells into nerve cells and glial cells present them as a potential treatment for AD. Successful evidence of action in animal models along with positive results in preclinical studies further encourage its utilization for AD treatment. With regard to humans, cell replacement therapy involving mesenchymal stromal cells, induced-pluripotent stromal cells, human embryonic stromal cells, and neural stems show promising results in clinical trials. However, further research is required prior to its use as stromal cell therapy in AD related disorders. The current review deals with the mechanism of development of anomalies such as Alzheimer’s and the prospective applications of stromal cells for treatment.

## Introduction

The epidemiological spread of communicable to non-communicable (not transfer from one living beings to another) diseases has raised the overall burden on healthcare workers worldwide. Neurological illnesses are serious issues that concern both medical professionals and patients when considering non-communicable diseases. Alzheimer’s disease (AD) accounts for 50%–70% of dementia along with it being the top fifth leading cause of death worldwide. By 2050, the worldwide population affected by AD is expected to rise to 152 million (GBD et al., 2019 Dementia Forecasting Collaborators, 2022). The degeneration of neural cells and synaptic connections in certain subcortical regions and cerebral cortex marks the presence of Alzheimer’s disease (AD). Scientists hypothesize that AD is based on cholinergic mechanisms, protein misfolding, and amyloid cascades. The loss of neural cells evident by temporal, parietal, frontal lobe atrophy, along with inflammation, increase in free radicals and the accretion of amyloid peptides and hyperphosphorylated tau protein in the form of plaques and neuro-fibrillary tangles (NFTs), respectively characterize Alzheimer’s disease (Valotassiou et al., 2010). An aggregation of misfolded proteins is the primary cause of this nervous system disorder. Two of these proteins are plaques (β-amyloid) and hyperphosphorylated tau proteins, which are created by the accumulation of Aβ proteins and affect cell-to-cell communication. These proteins also correspond to the formation of intracellular NFTs, which disrupt synaptic networks and reduce neurotransmitter production, respectively. Tangles, which are made of hyperphosphorylated tau protein, obstruct the neuronal internalised transport system that carries vital nutrients to the brain. The remaining 1% of the elderly population has been shown to have presenilin 2 (PS2) and presenilin 1 (PS1) genetic changes, which are responsible for reducing -secretase activity by boosting the synthesis of self-accumulating Aβ42 peptide. The diagnostic criteria for AD is given by the National Institute of Neurological and Communicative Disorders and Stroke (NINCDS) are: 1) the occurrence of disabilities related to cognition (language, orientation, learning and memory, perception behaviour), 2) the neuropsychological testing in case of symptoms concerning dementia syndrome confirmed by neuropsychological testing. There are no standard treatments presented in the literature (Loewenstein et al., 2001). However, the main strategy of treatment in AD reversal relies upon the reduction of Aβ levels. Various researchers across the globe have performed an extensive research to understand the pathogenesis, natural progression, diagnosis, and regenerative principles in the management of AD. However, “disease-modifying” treatments focusing on averting or curing the specific clinical observations and biomarkers related to AD still remain under investigation (Cummings and Fox, 2017). As per the complexities of Alzheimer’s pathophysiology, a multidisciplinary treatment approach is recommended composing of pharmacological therapy, behavioural treatment, and the stimulation of endogenous or exogenous neurogenesis and synaptogenesis (Doody et al., 2008; Liperoti et al., 2008; Radad et al., 2017). The present literature anticipates the current research advancements in stromal cell treatments of AD and discusses the challenges that still need to be addressed.

## Cellular therapy in Alzheimer’s disease

In 2004, the World Health Organization (WHO) introduced the idea of medical products of human origin (MPHO), which serve as a platform for the attainment and dispersal of biological therapeutic goods to treat a variety of diseases (Martin, 2017). The use of MPHO to treat diseases has become increasingly important as research in the realms of beneficial and developmental medicine along with pre-clinical examination has progressed. Regenerative medicine aims to rejuvenate at cellular and tissue levels while also achieving restored regulation in the regional milieu (Sharma et al., 2022). To regenerate the tissue of concern, regenerative medicine employs the recombination of the extracellular matrix, growth factors, and most importantly stromal cells.

The notion of “Cellular Therapy” (CT) or “Cytotherapy” is based on the use of paracrine signaling by biomacromolecules to direct stromal cells which are in a state of quiescence for induction or augmentation. CT attempts to induce a curative impact in the concerned area through the transplantation of autologous, allogeneic, or modified cells that are unaccompanied by changes in their biological properties. Recombinant cell-induced pluripotent cells, hematopoietic cells, genetically-modified cells, stromal cells, genetically designed cells, or an amalgamation of all of these cells can be used to create cellular therapeutic products (Kaufman, 2009; Nianias and Themeli 2019; Sharma et al., 2022). The use of human-made medical items to treat neurological problems is still in its early stages. For efficient regeneration of these cells, numerous combinations have to be researched and explored.

## Nervous stimulation of stromal cells

**FIGURE 1:**
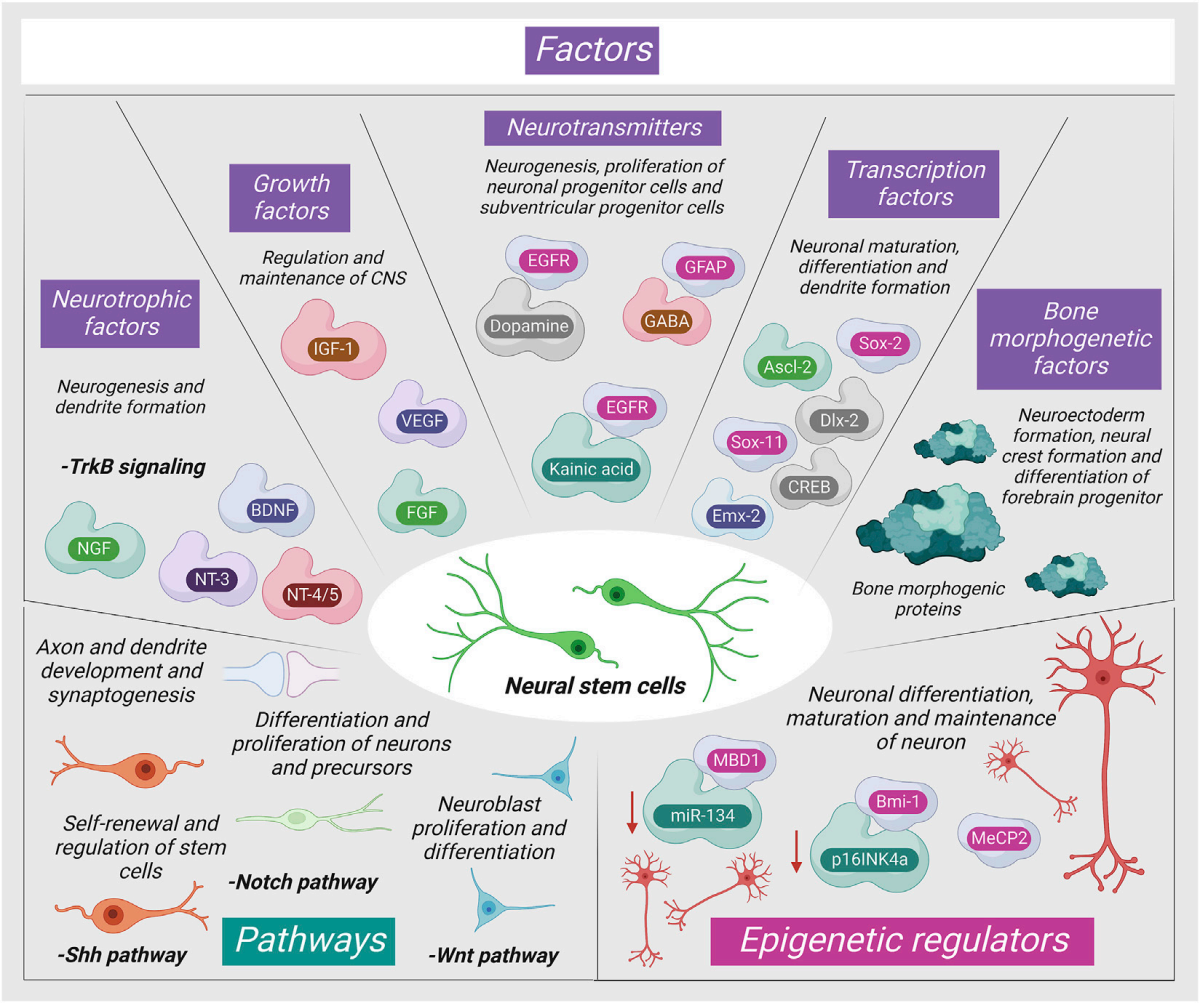
Mechanism of neurogenic signaling of stromal cells (MBD1: Methyl-CpG-binding domain protein one; NGF: Nerve Growth Factor; NT-3: Neurotrophin three; MeCP2: Methyl-CpG-binding protein two; BDNF: Brain-derived neurotrophic factor; IGF-1: Insulin-like growth factor; NT-4/5: Neurotrophin 4/5; VEGF: Vascular endothelial growth factor; MeCP2: Methyl-CpG-binding protein two; FGF: Fibroblast growth factor; Mll1: Mixed-lineage leukemia 1).

The adult stromal cells are stimulated for differentiation *via* numerous pathways as shown in Figure 1.

## Significance of stromal cells in Alzheimer’s disease

**FIGURE 2:**
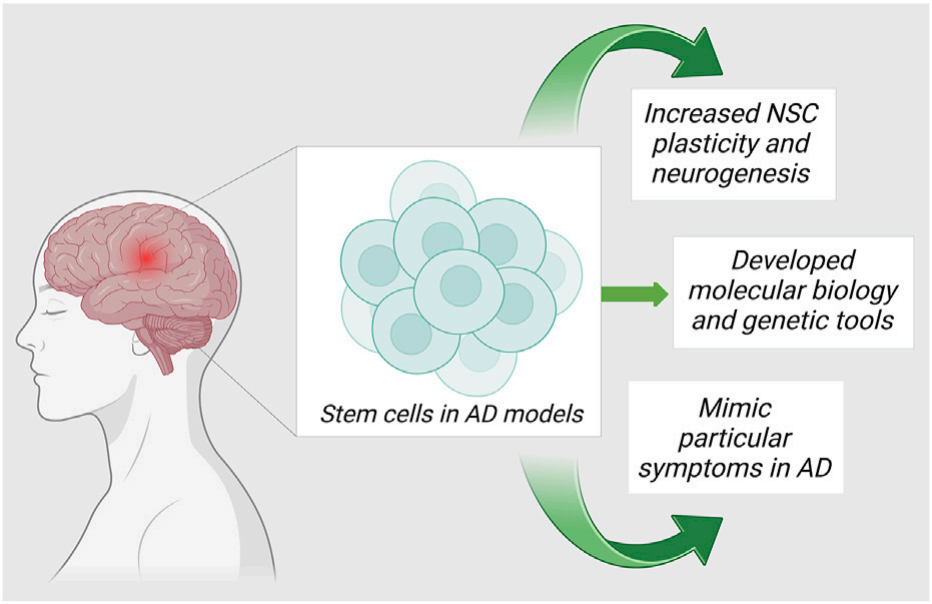
Strategies of stromal cell utilization in AD therapies.

Currently, the world lacks any proven standard therapy for the treatment of AD. Pharmacological management of AD temporarily improves cognitive symptoms. The targeted research in AD is directed towards 1) anti-oxidation, 2) removal of Aβ accumulation from the brain, and 3) regulation of the tau protein phosphorylation. Due to the advancements in regenerative and translational medicine, the usage of cells for treatment has become the limelight for producing disease-modifying strategies in the treatment of AD. Stromal cells are blank, unspecialized cells that are the result of the replenishment of lost neuronal cells through the differentiation and proliferation of neural cells upon activation *via* signaling pathways. They essentially restore neuroplasticity and neuromodulation (Ottoboni et al., 2020). In the recent decade, there are evidence to validate the cell-based neuroreplacement treatments for the treatment of AD in pre-clinical and clinical trials. The strategies of stromal cell therapy in AD are depicted in Figure 2. Evidence of the results from successful stromal cell therapy in animal models for AD is tabulated in Table 1.

**TABLE 1:** Laboratory trials of stromal cells in the treatment of AD.

Stromal cells	Animal model used	Results/Outcome
NSCs	Transgenic mice with triple mutation (3xTg-AD) expressing PS-1, APP, and tau	Reduced memory deprivation associated with position, location and learning along with increase in synaptic density
NPCs	Focal cerebral ischemia in rat model	Interaction between transplanted and endogenous cells was focussed;
		Enhanced intrinsic neural growth
UC-MSC	Transgenic mice with double mutation of APP and PS1	Microglial activation improves spatial learning and memory; Diminished accumulation of Aβ
Transdifferentiated human Wharton’s jelly MSCs into neuron-like cells	Transgenic mice with double mutation of AβPP/PS1	Development of perceptual observation
		Decrease in Aβ through expression of neprilysin and enzymes and activation of microglial cells
MSCs	A*β* treated mice	Enhanced neural tissue formation in hippocampus; Formation of neurons *via* Wnt signalling pathway from neural progenitor cells
BM-MSC	APP/PS1 transgenic mice	Decrease in accumulated plaques
		Escalated expression DeltaNp73 protein
		Improvement in spatial learning
Human olfactory bulb NSCs	Ibotenic acid induced AD rat model	Transcription of nerve growth factors
		Improved psychological abilities
P-MSCs	Aβ1-42 peptide infused in mouse model	Improved psychological abilities
		Increases neural cell development
VEGF overexpressing BM-MSCs	Transgenic mice with double mutation of APPswe/PS1dE9	Improvement in behaviour associated symptoms
		Decrease in amyloid plaque build-up
		Improvement in abnormal blood vessels formation
Choline acetyltransferase expressing hNSC	AF64A-cholinotoxin induced learning deficit rat model	Translocation of implanted cells to the damaged area; differentiation of neural stromal cells
ENCSCs	Aβ1-40 administration in rat	Increased granule cells in hippocampus; Expression of neuronal markers by implanted cells
UC-MSC	APP/PS1 transgenic mice model	Enhanced intrinsic expression of neprilysin
		Decrease in Aβ plaques

NSC, neural stromal cells; HNSC, human neural stromal cells; NPC, neural precursor cells; MSC, mesenchymal stromal cells; BM-MSC, bone marrow derived mesenchymal stromal cell; ENCSC, epidermal neural crest stromal cell; UC-MSC, umbilical cord-derived mesenchymal stromal cells; P-MSCs, placenta derived mesenchymal stromal cells.

Neurogenic precursors or neural stromal cells exist in a few restricted areas of the adult brain which shows continued neurogenesis and neuromodulation. Growth and the divergence of the cell into neurons and synapses are regulated by transcription factors and signaling pathways which involve biomolecules that act on neuronal progenitor cells, referred to as neurotransmitters. The genetic modification of neural stromal cells increases migratory efficiency. As a result, it allows to successfully employ them for the enhancement of gene expression and for the delivery of neurotrophic factors which subsequently modify the progression of AD.

### Embryonic stromal cells

Embryonic stromal cells (ESCs) are totipotent cells that have a self-renewing ability and differentiation capacity. ESCs are obtained from blastocysts, specifically the inner cell mass. Due to the pluripotent nature of ESCs, tumorigenesis, uncontrolled cellular explosion, and immunogenic rejection may occur. Tang et al. suggested that hippocampal ESC transplantation can be performed without any potential risks to restore cognitive function in Aβ peptide injured rats (J et al., 2008). In this rodent model of AD, ESC-derived neural progenitor cell (NPC) transplantation following commitment to a cholinergic cell phenotype can enhance behavioral and cognitive recovery with the regeneration of cholinergic neurons (Fh et al., 2009). Transplantation in the mouse with ESC-derived NPCs to the meynert basal nucleus resulted in enhanced memory and learning capacity (Liu et al., 2020). Along with that, the enhancement of neurocognitive recovery was also observed when mouse and human ESC-derived basal forebrain cholinergic neurons were transplanted into the transgenic AD mice model (Yue and Jing, 2015). ESCs derived from humans were able to translate into dopaminergic neurons, spinal motor neurons, and astroglial cells (Lee et al., 2007). Neural progenitor cells derived from ESC develop into neuron-like cells and astrocytes which enhances memory and learning performance in AD animal models (J et al., 2008). However, certain ethical concerns are associated with hESCs (Human ESCs) in FDA-approved clinical trials (Liras, 2010; Fouad, 2019). Estimating the effectiveness of transplantation-based therapy is crucial from an ethical standpoint, as is lowering the possibility of therapeutic misunderstanding, minimizing discomfort, and emphasizing the significance of informed permission. Actually, the ethical discussion around stem cell-based therapy demonstrated the challenging balance between the need for prudence and the advancement of clinical studies. For instance, the infinite and unwanted differentiation potential of iPSCs increases the possibility of cancer, human cloning, and the unethical creation of genetically engineered human embryos. Similar safety concerns arise with MSC implantation due to their propensity to promote tumour development and metastasis. As a result, iPSCs are viewed as having higher moral standing than hESCs. However, the greatest safety concern with iPSC-based treatment is the possibility of teratoma development because of the unregulated differentiation. Additionally, the proliferative and differentiation capacity is decreased due to the host cells’ niche differing from that of the *in vitro* cultivated cells.

### Mesenchymal stromal cells

MSCs play a significant role in controlling the symptoms of AD through 1) immunoregulation, 2) neurotrophic, 3) neuroplasticity, and 4) the reduction of Aβ plaque burden which restores the cognitive function (Babaei et al., 2012; Qin et al., 2020). Both *in vitro* and *in vivo*, MSCs show a remarkable capacity to trigger the quick clearance of Aβ aggregates. In terms of mechanism, the interaction between microglial cells and MSCs was primarily responsible for the removal of Aβ deposits (Fouad, 2019). Additionally, by expressing CCL5, MSCs can draw more microglial cells from the bone marrow (Fouad, 2019). In this approach, MSCs can inhibit deposition of Aβ plaques and perhaps halt the progression of the illness. Intriguingly, these investigations also showed a decrease in intracellular NFTs. Various preclinical studies have demonstrated the reversal of pathological changes in AD animal models. These studies verified neuroprotection, neuroplasticity, neuromodulation, and the neurogenic effects of MSCs through the neurogenic signaling pathway activation (Fumagalli et al., 2006). These grafted MSCs upregulate the expression of the anti-apoptotic factor, accentuate the AD symptoms, and halt the progression of the AD (Ruzicka et al., 2016). MSC-induced cognition restoration is due to the suppression of microglial activation and reduced levels of Aβ plaques in the brain (Woodruff-Pak, 2008).

The administration of bone marrow-derived MSCs (BM-MSCs) in the intracerebroventricular region ameliorates memory loss in AD model mice by accentuating astrocytic inflammation as well as synaptogenesis. BM-MSCs treated AD model of rodents expressed increased levels of miR-146 exosomes in the hippocampus which ultimately induces synaptogenesis and neurogenesis. Along with that, neuroplasticity was restored and cognitive impairment improved (Nakano et al., 2020). Intravenous injection of BM-MSCs was detected in brain parenchyma within an hour post-injection in the rat AD model which ultimately released growth factors and cytokines to recover neurobehavioral functions and stimulate endogenous regeneration (Harach et al., 2017).

Endovenous introduction of MSCs derived from adipose tissue (AD-MSCs) in rodent-model with AD improved neural functioning. It was accompanied by an increase in the formation of neurons along with differentiation of cells resembling neurons and astrocytes. Advancement in cognitive abilities was evident due to the decrease in accumulation of amyloid plaque and the increase in neural cells. The cells were then observed 12 days after injection (Tian et al., 2012).

Intracerebroventricular transplantation of placenta-derived MSCs (PD-MSCs) demonstrated the inhibitory effect of neuronal apoptosis and memory impairment in Aβ_1–42_-infused mice (Yun et al., 2013). PD-MSCs decrease the cytokines with pro-inflammatory action and increase the concentration of anti-inflammatory cytokines in the brain. They promote neuronal differentiation and proliferation in the hippocampus of the AD mice model. MSC transplantation in aged AD rats restores motor and cognitive activity (Liu et al., 2020). There are encouraging results in the preclinical trials of MSCs in AD. In one human trial, composed of nine patients, were administered MSCs in AD and have completed phase I which confirms the potential, efficacy, and non-toxicity of MSC injection.

### Neural stromal cells

The multipotent nature of neural stromal cells (NSCs) allows them to differentiate into oligodendrocytes, astrocytes, and neurons after transplantation which is why they are an ideal cell-based substitution therapy in degenerative neurological disorders. The sources of NSCs are from primary tissues namely fetus, iPSCs, and ESCs. However, the required NSCs have to be customized for a particular patient to avoid immune rejection. Moreover, the isolation procedure is extremely risky as well. As contrary to other stem cells like ESCs, foetal NSCs, and MSCs, adult neural stem cells (aNSCs) are localised to certain regions of the central nervous system (CNS) and have a low proliferative potential. As a result, the initial isolation and steady *in vitro* growth of aNSCs are significant technological challenges that must be overcome before aNSCs may be used. Adult CNS surgical samples are often comparatively small (1–2 ml). Since there aren't many resident aNSCs in the tissue, isolation methods have been improved to increase the likelihood that aNSCs will be successfully isolated in the first place. CNS tissues are manually chopped and enzymatically degraded into single cells to get aNSCs. Enzymatic digestion is one among them, and it is important because it directly impacts aNSC survival. Different researchers use different dissociating enzyme compositions and incubation periods. Trypsin, collagenase, and papain have often been utilised, and in some studies, papain dissociation was recommended as the most effective method for the first separation of aNSCs. In the mammalian brain, NSCs exhibit neural homeostasis, repair, and regeneration, and demonstrate neural pleiotropism (Boese et al., 2020).

Administration of both stromal cells and neurotrophic peptides, stimulates differentiation of the neural stromal cells *via* paracrine signaling. The mentioned peptides increase neural formation in zones of the brain that concern memory such as the subgranular and subventricular zone. These neural stromal cells differentiate into neuronal cells and glial cells (Shohayeb et al., 2018). Genetic engineering of these cells amplifies the release of amyloid plaque degrading enzymes which increases synaptic density by removing Aβ aggregates (Hayashi et al., 2020). The immune protection of MSCs can be provided by the capacity to regulate immune activities thus providing neural protection from inflammation. Aside from that, mi-RNAs and si-RNAs released from these MSCs act to degrade accumulated amyloid plaques.

Transplantation of neural SCs in the lateral ventricle resulted in the formation of neuronal progenitor cells and glial cells. The cells had the capability of migration and regeneration which reduced Aβ deposition and tau phosphorylation and increased neural cell and synapse formation (Hayashi et al., 2020). Findings were proven through the incorporation of fluorescent protein labeled BM-MSCs in the hippocampus of AD diagnosed models where the same results were witnessed (Duncan and Valenzuela, 2017). Human MSCs were able to restore neuromuscular, cognition, and memory function after administration in aged rat models.

Transplanted NSCs in AD animal models possess the ability to replace the destructed neural circuit through neurotrophic factors that counteract the levels of Aβ plaques. NSC transplantation improve the cholinergic neuron numbers and enhance learning and memory abilities (Hayashi et al., 2020). It is unclear whether the observed behavioral rescue, enhanced memory, and learning abilities are due to NSC differentiation or neurotrophic factors in AD models (Hayashi et al., 2020). However, transplanted grafts of Neural SCs increases neurotrophic factor levels that are derived from the brain and enhance the behavioral rescue without altering concentration concerning Aβ or tau in a mutant rodent model of AD with overexpressed hAPP (Marsh and Blurton-Jones, 2017).

In APP/PS1 transgenic mice AD model, intranasal incorporation of human NSCs that differentiate into cholinergic neurons decrease *β*-amyloid accumulation by increasing neprilysin expression along with enzymes that can degrade *β*-amyloid. They also downregulate neuroinflammation, synaptic and pericytic loss ultimately rescuing the cognitive function. Intranasal administration of human NSCs possess greatest potential as a noninvasive tool in treating AD (Lu et al., 2021). NSCs ameliorates Aβ levels in AD mouse models by degrading plasmin, cathepsin B, and insulin-degrading enzymes.

Zhang et al. (2019) transplanted human induced neural progenitor cells (iNPCs) into the hippocampal region of brain in wild type immunodeficient AD mice. This study demonstrated the long-term survival, graft-host synaptic connections, neuronal circuits, and regenerated neural network of host hippocampus. AD mice exhibited neuroplasticity, neuromodulation and synaptogenesis with enhanced cognitive and behavioral abilities.

In APP/PS1 murine model of AD, NSC infiltration into fimbria and fornix junction show significant improvement in cognition and memory abilities. NSCs induce microglia activation and phagocytosis of amyloid plaques (McGinley et al., 2018). Without priming the microenvironment with neurotrophic factors, the transplantation of NSCs to the hippocampus would not produce any desired results in AD. In aged animals, the infusion of NGF demonstrated improved cognition. In phase one of the clinical trial, genetically modified fibroblasts coded with NGF gene transplantation into forebrain found to be safe (NGF-gene therapy) for AD (Tuszynski et al., 2005). These evidence provide a right path and new horizon in the management of AD in humans.

### Induced pluripotent stromal cells

In 2011, Yahata et al. and Yagi et al. were the first researchers to introduce iPSCs therapy for AD (Yagi et al., 2011; Yahata et al., 2011). Yahata et al. (2011) demonstrated the production of Aβ plaques in neurons through the transplantation of iPSCs from forebrain neurons. Yagi et al. (2011) demonstrated the enhanced Aβ42 secretion by attenuating the dysfunctional condition of familial AD with PS1 (A246E) and PS2 (N141I) mutations.

Induced pluripotent cells can transform into neurospheres, neuronal progenitor cells and, neural cells which is evident in the cortex of rodent. The incorporation of these cells in diseased rodents resulted in increased concentration of Aβ-degrading protease neprilysin (Han et al., 2019). Enhanced synaptic activity and the connection between excitatory and inhibitory neurons have been demonstrated by iPSC-derived neurons (Birey et al., 2017; Xiang et al., 2017). Pure, immature neuronal cells, after incubation, differentiated into mature nerves exhibiting the inhibitory GABAergic and excitatory glutamatergic synapses along with the potentiation of action potentials. Human iPSC derived cortical neuronal cells attenuate the deposition of Aβ plaques and tau protein complexes in the forebrain (Nieweg et al., 2015).

Treatment of AD models by AD-iPSCs derived from neuronal cells consequently suppressed p-tau protein. AD-iPSC derived neuronal cells were able to upregulate AD associated genes which was evident by increased transcription (Hossini et al., 2015). In 5XFAD transgenic AD mouse model, differentiation of protein-iPSCs into glial cells, attenuation of Aβ plaque depositions, and improved cognitive ability were observed. In proteomic analysis, upregulation of oligodendrocyte-related genes in brain when protein-iPSCs are injected were observed (Cha et al., 2017).

Three dimensional spheroids from human iPSCs for AD model were differentiated into neuronal cell lines that led to the reduction of both Aβ40 and Aβ42 thereby potentiating the cell lines to regenerate into neuronal cells (Lee et al., 2016). Three dimensional cerebral organoids recapitulated the human brain architecture including cerebral progenitors in the subventricular and subgranular zone of hippocampus (Machairaki, 2020). Human iPSCs yielded 16 times more extracellular vehicles, exosomes, than MSCs. These exosomes have more potential to attenuate the deposition of Aβ plaques in the brain and demonstrate an increased Aβ42/Aβ40 ratio in AD animal models (Machairaki, 2020).

## Clinical trials

### Challenges posed by stromal cell treatment in Alzheimer’s disease

The emerging application of clinical therapy by medical experts and researchers in neurological disorders is based on the proof of potential in the preclinical research. The methods discussed have proven to be efficient and safe for the treatment of neural medical conditions. Following preclinical trials, clinical trials (Table 2) have begun to specifically target AD (McGinley et al., 2016). Although certain challenges still obstruct the routine use of cellular therapy in the medical practice, it is important to identify the correct cell types that are used along with understanding the mechanism of action of those cells for a maximum advantage in treatment. For instance, the therapy for treatment of PD requires particular cells such as the dopaminergic substantia nigra neurons (Freed et al., 2001).

**TABLE 2:** Clinical trials of Alzheimer’s disease incorporating stromal cells.

S.No.	Nct number	Title	Status	Interventions	Locations
1	NCT02833792	Allogeneic Human Mesenchymal Stromal cells for Alzheimer’s Disease	Recruiting	BMMSCs	United States
2	NCT02600130	Allogeneic Human Mesenchymal Stromal cell Infusion Versus Placebo in Patients With Alzheimer’s Disease	Active, not recruiting	BMMSCs	United States
3	NCT04040348	Alzheimer’s Disease Stromal cells Multiple Infusions	Recruiting	BMMSCs	United States
4	NCT03117738	A Study to Evaluate the Safety and Efficacy of AstroStem in Treatment of Alzheimer’s Disease	Completed	ADMSCs	United States
5	NCT02672306	Safety and Exploratory Efficacy Study of UCMSCs in Patients With Alzheimer’s Disease	Unknown status	UCMSCs	China
6	NCT04855955	Autologous Human Adipose-Derived Mesenchymal Stromal cells in Alzheimer’s Disease	Available	ADMSCs	United States
7	NCT03724136	Alzheimer’s Autism and Cognitive Impairment Stromal cell Treatment Study	Recruiting	BMMSCs	UAE
8	NCT01696591	The Long-Term Safety and Efficacy Follow-Up Study of Subjects Who Completed the Phase I Clinical Trial of NeurostemÂ®-AD	Unknown status	ADMSCs	Korea
9	NCT01547689	Safety and Efficiency of Umbilical Cord-derived Mesenchymal Stromal cells (UC-MSC) in Patients With Alzheimer’s Disease	Unknown status	UCMSCs	China
10	NCT02054208	Safety and Exploratory Efficacy Study of NEUROSTEMÂ^®^ Versus Placebo in Patients With Alzheimer’s Disease	Completed	UCMSCs	Korea
11	NCT04482413	Study to Evaluate the Safety and Efficacy of AstroStem in Treatment of Alzheimer’s Disease	Not yet recruiting	ADMSCs	United States
12	NCT01297218	The Safety and The Efficacy Evaluation of NEUROSTEMÂ®-AD in Patients With Alzheimer’s Disease	Completed	UCMSCs	Korea
13	NCT04954534	Exploratory Efficacy Study of NEUROSTEMÂ^®^ in Subjects Who Control Group of NEUROSTEMÂ^®^	Not yet recruiting	UCMSCs	Korea
14	NCT04684602	Mesenchymal Stromal cells for the Treatment of Various Chronic and Acute Conditions	Recruiting	ADMSCs	United States
15	NCT03172117	Follow-up Study of Safety and Efficacy in Subjects Who Completed NEUROSTEMÂ^®^ Phase-I/IIa Clinical Trial	Recruiting	UCMSCs	Korea
16	NCT03297177	Autologous Stem/Stromal Cells in Neurological Disorders and Disease	Not yet recruiting	ADMSCs	United States
17	NCT00874783	Development of iPS From Donated Somatic Cells of Patients With Neurological Diseases	Recruiting	iPSCs	Israel

Similarly, vascular endothelial cells, neuronal cells, and glial cells are needed for therapy which aims to treat AD and traumatic brain injuries. Considering this, there is a need of specifically differentiated cell lines. Known mechanisms today act through paracrine signalling which involves neurotrophic factors that regulate immune functions. Another potential mechanism could be inducing neural cell formation (Gnecchi et al., 2008). Altogether, it is critical to comprehend these effector processes before using them to treat neurological illnesses.

Results expressing affectivity of stromal cells in animal models are not completely reliable due to numerous reasons. The incomplete aspects and inaccuracy may have more harmful outcomes than beneficial. First being, using rodent models are far from the accurate representation of the physiological state of sickness in humans. Second, the age variation between the two remains at an excessive gap. Third, animal models are still inadequate to show that clinically relevant functional impairments improve. Finally, the animal models may be incapable of indicating the negative consequences of CT (Van der Worp et al., 2010).

The characterization of potential tumour generation remains the next issue in CT. For the treatment of neurological disorders, a healthy average life span and the tumorigenicity potential is important. In this circumstance, even a remote potential of tumour growth through CT would be unbearable. As a result, substantial research into the tumour formation of stromal cells and the derivatives of stromal cells is critical. In order to avoid risk of such terrible incidents and their associated consequences for patients, researchers and healthcare specialists must work together. Furthermore, treatments must be effectively better in terms of safety and effectiveness when compared to conventional therapeutic interventions (Sato et al., 2019).

Stromal cell treatments are linked to moral and ethical concerns along with the possibility of transplant rejection. With these cellular therapies, the cost of customized treatment escalates as a result of poor efficiency in the elicitation of iPSCs from the patient’s diploid cells. In order to increase the effectiveness of these cellular therapies while minimizing cost, technical and methodological improvements are required. The use of SCs in AD treatment raises a lot of unresolved issues. There are no data on which sort of differentiated or undifferentiated pluripotent SCs would be the most efficient for this treatment. Likewise, details on cell concentration, the quantity of effective dosages, and the length of the course of treatment are lacking. It is unclear if a confined (using stereotactic injections) or peripheral transplant should be performed, as well as how cell migration to the afflicted part of the brain happens. There is no proof that SCs treatment works in humans. It is also unknown how SCs can get rid of the inflammatory and toxic environment caused by the Aβ peptide, how healthy cells connect to one another, or how the cell repopulation will influence other metabolic pathways. It is yet unclear if the transplantation of SCs will stop the transmission of pathogenic tau protein between neurons or communication between damaged cells. Finally, since it has been unable to show the safety of this form of therapy for AD, ongoing patient monitoring would be necessary to prevent any negative side effects, both immediate and long-term.

Many SCs treatments are currently in the testing phase. Although SCs products may have a huge potential to cure a wide range of medical conditions, there is not enough scientific proof to guarantee that their usage is safe and has any positive health effects. Despite the fact that SCs have been the subject of several research in animal models of AD, the translation of this approach to the treatment of AD has been hampered by the differences between the molecular patterns of these models and those of people, as well as the conflicting results achieved. Before the advantages of this therapy can be demonstrated in the clinic, further in-depth understanding of the cellular pathways relating to its usage in both animal models and people is required.

## Conclusion

In the absence of robust treatment methods for AD, research on SCs seem to be a major prospect. The variety of SCs, iPSCs, MSCs, ESCs and NSCs, can be incorporated invasively and non-invasively. This presents researchers with a wide range of possibilities for the development of customized cellular therapies. The promising results observed in pre-clinical and clinical trials were evident of the decrease in causative agents of AD which encourages its use in humans. Nevertheless, enormous amounts of research are requirement especially in the context of genetic variation due in the occurrence of AD and the inability to conclude negative consequences due to lack of replicable translation models. Issues like accuracy of treatment, mechanistic methodology, and ethical issues also need to be addressed prior to its common medical use.

Furthermore, there are notable changes in the brain’s structure and microenvironment between AD patients and animal models, making it challenging to precisely characterise the positive effects of stem cells in human AD. For the familial form of AD, transgenic animal models have been created, although human AD pathogenesis primarily comprises sporadic instances. This restricts our understanding of how patient-specific stem cell treatment might operate. These issues require future research. Future preclinical studies examining various stem cell sources, kinds, dosages, long-term safety, effectiveness, and exact mechanisms of action are necessary. Using recently developed *in vivo* biomarkers in clinical research is anticipated to give researchers more control and insight into patient selection and quantity efficacy than was previously feasible using previously known marker kinds. When working with the immune system, neuro factors, enzymes, proteins, and gene therapy, to mention a few examples of applications, among many more, cell extraction and injection enhancements may be tackled from numerous viewpoints. Additionally, as part of the entire development process for the technology, it is crucial to evaluate ethical issues at each stage of building a stem cell-based technology.
